# Supplementary material for: Risk Factors for 30-Day Mortality in Nosocomial Enterococcal Bloodstream Infections
Source: Antibiotics (Basel). 2024 Jun 27;13(7):601. doi: 10.3390/antibiotics13070601 (PMC11273391; doi:10.3390/antibiotics13070601)
Supplement: Supplementary file 1 [file antibiotics-13-00601-s001.zip › antibiotics-3042266-supplementary.pdf]

antibiotics

Supplementary Materials

File S1. Appropriate Therapy

The following antimicrobial regimens have been considered appropriate for *E. faecalis* bacteremia: vancomycin (65), linezolid (7), teicoplanin (3), daptomycin, amoxicillin/clavulanate (22), piperacillin/tazobactam (69), ampicillin (13), imipenem (7), ampicillin plus gentamicin (6), ampicillin plus ceftriaxone (30), ampicillin plus cefepime, daptomycin plus ampicillin (1), tigecycline (1).

The following antimicrobial regimens have been considered appropriate for VS *E. faecium* bacteremia: piperacillin/tazobactam (6), vancomycin (40), teicoplanin (1), tigecycline, linezolid (7), daptomycin, daptomycin plus ampicillin (1).

For VR *E. faecium* BSI we considered as appropriate the following regimens: daptomycin (4), daptomycin plus ampicillin (6), daptomycin plus intravenous fosfomycin (1), linezolid (13), tigecycline (1), tigecycline plus intravenous fosfomycin (1).

N.b. The frequencies of treatments are indicated in parentheses.
